# The MIF promoter SNP rs755622 is associated with immune activation in glioblastoma

**DOI:** 10.1172/jci.insight.160024

**Published:** 2023-07-10

**Authors:** Tyler J. Alban, Matthew M. Grabowski, Balint Otvos, Defne Bayik, Wesley Wang, Ajay Zalavadia, Vlad Makarov, Katie Troike, Mary McGraw, Anja Rabljenovic, Adam Lauko, Chase Neumann, Gustavo Roversi, Kristin A. Waite, Gino Cioffi, Nirav Patil, Thuy T. Tran, Kathleen McCortney, Alicia Steffens, C. Marcela Diaz, J. Mark Brown, Kathleen M. Egan, Craig M. Horbinski, Jill S. Barnholtz-Sloan, Prajwal Rajappa, Michael A. Vogelbaum, Richard Bucala, Timothy A. Chan, Manmeet S. Ahluwalia, Justin D. Lathia

**Affiliations:** 1 Department of Cardiovascular & Metabolic Sciences and Imaging Core, Lerner Research Institute,; 2 Center for Immunotherapy and Precision Oncology, and; 3 Rose Ella Burkhardt Brain Tumor and Neuro-Oncology Center, Cleveland Clinic, Cleveland, Ohio, USA.; 4 Nationwide Children’s Hospital, Institute for Genomic Medicine, Departments of Pediatrics and Neurological Surgery, The Ohio State University Wexner Medical Center, Columbus, Ohio, USA.; 5 Division of Cancer Epidemiology and Genetics, Trans-Divisional Research Program, Center for Biomedical Informatics and Information Technology, National Cancer Institute, Bethesda, Maryland, USA.; 6 University Hospitals Research and Education Institute, Cleveland, Ohio, USA.; 7 Yale School of Medicine and Yale Cancer Center, New Haven, Connecticut, USA.; 8 Departments of Pathology and Neurosurgery, Northwestern University, Feinberg School of Medicine, Chicago, Illinois, USA.; 9 Cleveland Clinic Lerner College of Medicine at Case Western Reserve University, Cleveland, Ohio, USA.; 10 Case Comprehensive Cancer Center, Cleveland, Ohio, USA.; 11 Departments of Cancer Epidemiology and Neuro-Oncology, H. Lee Moffitt Cancer Center, Tampa, Florida, USA.; 12 Miami Cancer Institute, Baptist Health South Florida, Miami, Florida, USA.

**Keywords:** Immunology, Oncology, Brain cancer, Cellular immune response, Immunotherapy

## Abstract

Intratumoral heterogeneity is a defining hallmark of glioblastoma, driving drug resistance and ultimately recurrence. Many somatic drivers of microenvironmental change have been shown to affect this heterogeneity and, ultimately, the treatment response. However, little is known about how germline mutations affect the tumoral microenvironment. Here, we find that the single-nucleotide polymorphism (SNP) rs755622 in the promoter of the cytokine macrophage migration inhibitory factor (*MIF*) is associated with increased leukocyte infiltration in glioblastoma. Furthermore, we identified an association between rs755622 and lactotransferrin expression, which could also be used as a biomarker for immune-infiltrated tumors. These findings demonstrate that a germline SNP in the promoter region of *MIF* may affect the immune microenvironment and further reveal a link between lactotransferrin and immune activation.

## Introduction

Well-known somatic drivers of glioblastoma (GBM) heterogeneity, including *PDGFRA*, *IDH1*, *EGFR*, and *NF1*, have altered how we treat patients, diagnose disease, and design clinical trials (1, 2). While many groups have sought to identify germline variants associated with disease prevalence and outcomes, it is still unclear how germline variants alter the tumoral heterogeneity and immune microenvironment. Here we focus on understanding the tumoral effects of 1 well-characterized germline single-nucleotide polymorphism (SNP), rs755622, which has been shown to be associated with increased expression of the cytokine macrophage migration inhibitory factor (MIF) (3–20). MIF is an important microenvironmental factor in glioma, and we previously identified an immune-suppressive pathway in GBM that is driven by secreted MIF from cancer stem cells (CSCs) that in turn activates myeloid-derived suppressor cells (MDSCs) (21). Recent work from our laboratory and others has shown that MDSCs are increased in the circulation and tumor microenvironment (22), they portend a poor prognosis (23), their expansion can be driven by CSC-derived MIF (21, 23), and they can be reduced by MIF neutralization (either genetically or pharmacologically) (24, 25). Furthermore, *MIF* has been studied in a variety of cancers in the context of inflammation and has been found to regulate immune activity (3–20).

*MIF* expression is influenced not only by disease state but also by functional germline genetic polymorphisms. The most notable is a *MIF* promoter SNP, rs755622, which has been associated with multiple inflammatory conditions such as lupus, rheumatoid arthritis, septic shock, and cardiovascular disease (3, 5, 8, 13, 15). This *MIF* SNP is in linkage disequilibrium with the presence of 7 CATT repeats at the –794 promoter microsatellite, which leads to tighter binding by the transcription factor ICBP90 when compared with the more commonly occurring 5 or 6 CATT repeats (26). Accordingly, rs755622 is commonly analyzed in place of the –794 CATT microsatellite (15). While the minor allele frequency of the rs755622 *MIF* SNP ranges from 15% to 20% in White individuals and > 45% in individuals of African descent, it has not been associated with GBM growth or survival in large-scale genome-wide association studies (27). Given the role of MIF in immune response, we hypothesized that this *MIF* SNP may be associated with the immune microenvironment of GBM. Here, we determined that patients with the *MIF* SNP rs755622 have an altered tumor microenvironment characterized by increased lymphocyte infiltration and enhanced lactotransferrin (*LTF*) expression. Furthermore, high *LTF* expression can serve as an indicator for a subset of tumors that have high lymphocyte infiltration.

## Results

### Patients with the MIF SNP rs755622 have an increase in LTF expression and increased immune microenvironment signaling.

**Figure 1:**
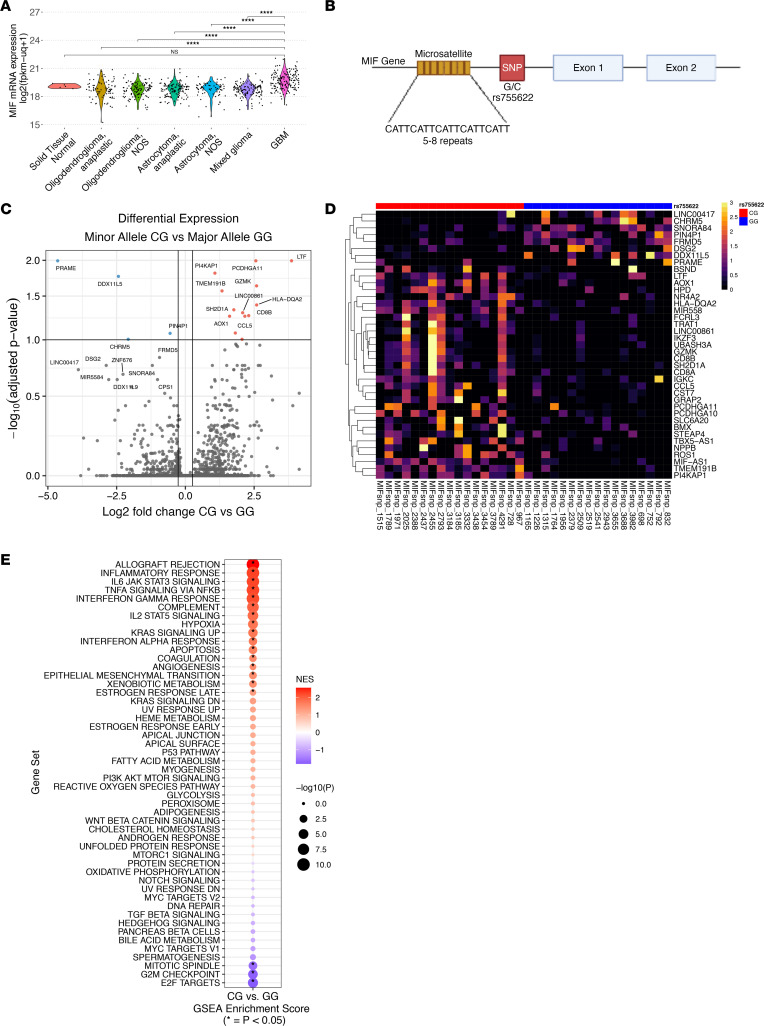
Patients with the *MIF* SNP rs755622 have an increase in lactotransferrin (*LTF*) and immune microenvironment signaling. (**A**) Expression of the *MIF* gene mRNA in TCGA_GBMLGG (*n* = 701) was compared among the histological subtypes astrocytoma, oligodendroglioma, oligoastrocytoma, and GBM using unpaired *t* test. (**B**) The *MIF* gene structure highlighting the -794 CATT repeat, which contains between 5 and 8 repeats, and the *MIF* SNP rs755622 at position -173. (**C**) Using GBM samples from (*n* = 17) patients with a G/G genotype and (*n* = 17) with a C/G genotype, differential expression analysis was performed using DESeq2 on raw counts and the genes with log fold-change > 1 and with *P* > –log_10_ (adjusted *P* value). (**D**) The 25 genes with the largest increases and decreases in gene expression by fold change are shown via heatmap (rs755622; red, CG; blue, GG; ID, colors indicate individual samples with column being a unique sample). Heatmap rows are clustered using hierarchical clustering; transcripts per million are scaled to rows for heatmap color scale. (**E**) GSEA was performed using Hallmark gene sets on the differentially expressed gene list comparing the C/G genotype to the G/G genotype sorted by log_2_ fold-change for gene-rank position. Normalized enrichment scores (NES) are shown in red/blue, with red enriched in C/G and blue enriched in G/G, while the size of the circle represents the –log_10_ *P* value as determined by GSEA.

Based on our previous assessments of *MIF* as a driver of CSC/MDSC-mediated communication (21), we assessed overall *MIF* expression levels across brain tumors and found elevated MIF in isocitrate dehydrogenase (*IDH*) WT GBM patient tumor samples when compared with patients with lower-grade (*IDH* mutant astrocytomas and oligodendrogliomas) gliomas and normal tissue using TCGA data (Figure 1A). Based on these information, we hypothesized that patients with GBM may have an increased prevalence of the regulatory *MIF* rs755622 nucleotide -173 G/C SNP (Figure 1B), which has been shown to be 1 factor with the ability to increase *MIF* expression. We assessed the rs755622 *MIF* SNP across 3 separate, annotated clinical cohorts of patients with GBM (total of 966 patients, including 449 from Cleveland Clinic, 386 from Moffitt Cancer Center, and 131 from Case Western Reserve University/University Hospitals of Cleveland). Our analysis of individual and combined cohort statistics revealed significant differences in the frequency of key prognostic indicators among cohorts (Karnofsky Performance Score [KPS], total surgical resection, receipt of standard of care [SOC], and recurrence) across the 3 cohorts (Supplemental Figure 1, A and B; supplemental material available online with this article; https://doi.org/10.1172/jci.insight.160024DS1). Across all cohorts, we genotyped patients via PCR and found a similar frequency of *MIF* SNP rs755622 major (G/G) and minor (C/C or C/G, noted as C/*) allele-containing genotypes in each (Supplemental Figure 1C). Furthermore, these frequencies matched the expected frequencies reported in the 1000 Genomes Project in people of European ancestry (Supplemental Figure 1C). Previous reports (28) have also shown a strong linkage disequilibrium between the rs755622 SNP and the CATT repeats rs5844572, so we further assessed the CATT repeat length in patients with available DNA (Supplemental Figure 2, A and B). In this analysis, we found a > 90% cosegregation between CATT repeats 7-8 (rs5844572) and the *MIF* SNP (rs755622), in alignment with previous studies (28). When evaluated according to major clinical prognostic indicators for GBM, we observed significant associations of sex and receipt of SOC with the rs755622 *MIF* SNP genotype using descriptive analysis (Supplemental Figure 1, B, D, and E, and Supplemental Figure 3). Univariate and multivariable survival analyses did not demonstrate any differences in overall or progression-free survival (PFS) according to rs755622 *MIF* SNP genotype in any individual cohort or when data from all 3 cohorts were combined (Supplemental Figure 1, D–F, and Supplemental Figures 3 and 4).

When analyzing individual cohorts by univariate analyses and combined analyses, we observed no significant difference in GBM incidence or patient survival between the rs755622 *MIF* SNP genotypes; however, we hypothesized that there may be differences in tumor and microenvironment interactions between genotypes given the association of the rs755622 *MIF* SNP with inflammation in nononcologic conditions (Supplemental Table 1). To explore this possibility, we performed RNA-Seq on 17 patients with primary, untreated GBM from each MIF genotype in our Cleveland Clinic cohort who had similar clinical parameters and outcomes (Supplemental Table 2). In selecting this cohort, we included patients with good prognosis (greater than 6 months PFS from diagnosis) and poor prognosis (less than 6 months PFS from diagnosis). We balanced our data set in this way to ensure that we did not bias our results toward an immunologic phenotype based on observations that patients with better outcomes have enriched lymphocytes (22, 29, 30). Differential gene expression analysis revealed that the rs755622 minor allele patients (e.g., -173 C SNP) had an enrichment in immune cell–related genes, including *LTF*, *GZMK*, *HLA-DQA2*, *CD8B*, and *CCL5* (Figure 1, C and D).

**Figure 2:**
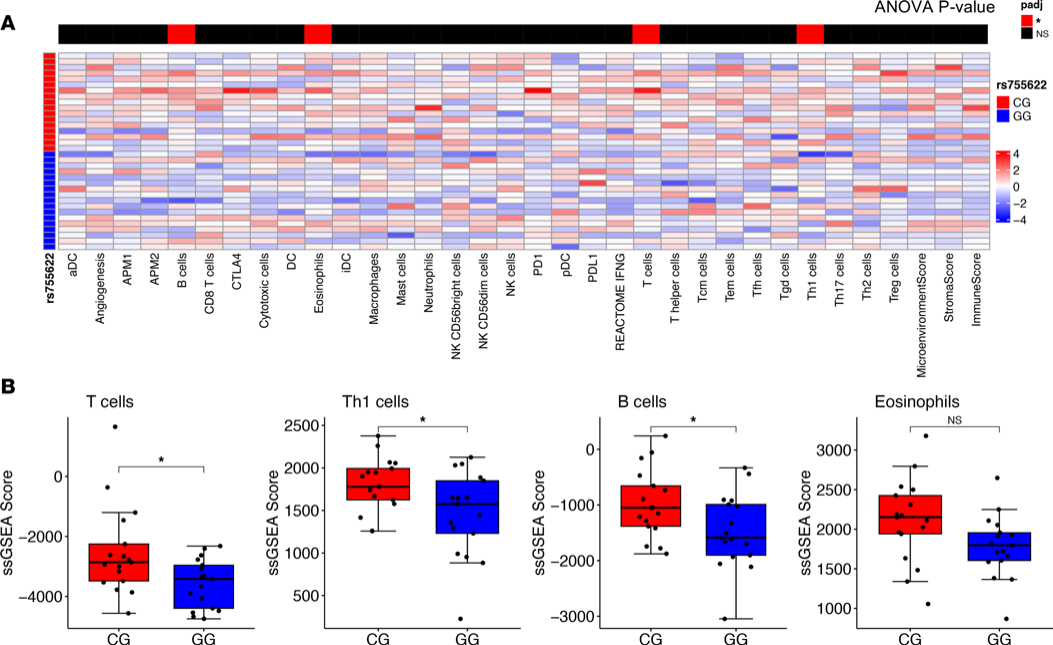
Patients with the *MIF* SNP rs755622 have an increased immune infiltration. (**A**) ssGSEA analysis was performed using the R package GSVA for cell type gene signatures, while xCell was utilized to estimate the overall immune, microenvironment, and stromal scores. ANOVA was performed to compare the deconvolution scores between patients with C/G and G/G genotypes, with the *P* value shown above the heatmap of the scaled scores. (**B**) ssGSEA scores for significant populations are shown as a box plot with unpaired *t* test *P* values shown for each.

Gene-set enrichment analysis (GSEA) utilizing Hallmark curated gene sets identified a significant increase in inflammatory pathways, including Interferon Gamma Response and Allograft Rejection, in *MIF* SNP minor allele–containing tumors (Figure 1E). Additionally, ingenuity pathway analysis (IPA) showed similar findings, with increased innate immune response and prostaglandin signaling among the top enriched pathways of patients with the *MIF* SNP minor allele (Supplemental Figure 5). In seeking to better understand the individual immune cell types that were different between genotypes, we used single-sample GSEA (ssGSEA) deconvolution with curated gene sets related to immune cells and immunotherapy. In these analyses, we observed a significant increase in cell signatures associated with an immune response in patients with the minor allele compared with those with the major allele; significance was calculated by 2-way ANOVA and is indicated by the *P* value bar above each column of the heatmap (Figure 2, A and B).

Of the differentially expressed genes, *LTF* was the most significantly upregulated in patients with the minor allele and positively correlated with increased T cell and immune responses (Figure 1C). LTF is considered a key factor in first-line immune defense against bacteria, yeast, viruses, fungi, and parasites and may additionally contribute to antitumor response (31–33). LTF is an acute-phase immune mediator released from neutrophils and participates in the switch from innate to adaptive immune response. LTF signals through TLRs in myeloid cells to activate NF-κB and CD40 expression and to promote the initiation of antitumor immune responses (34, 35). It has also been associated with M1 polarization and, in a pancancer study, negatively correlated with tumor mutational burden (36). Taken together, these analyses suggest that, while the rs755622 *MIF* SNP did not associate with differences in GBM incidence or survival, rs755622 did correlate with a difference in the immune cell composition of the tumor microenvironment.

### Immunofluorescence staining confirms enhanced T cell infiltration and CD8^+^ T cell activation in patients with GBM with the MIF SNP rs755622.

**Figure 3:**
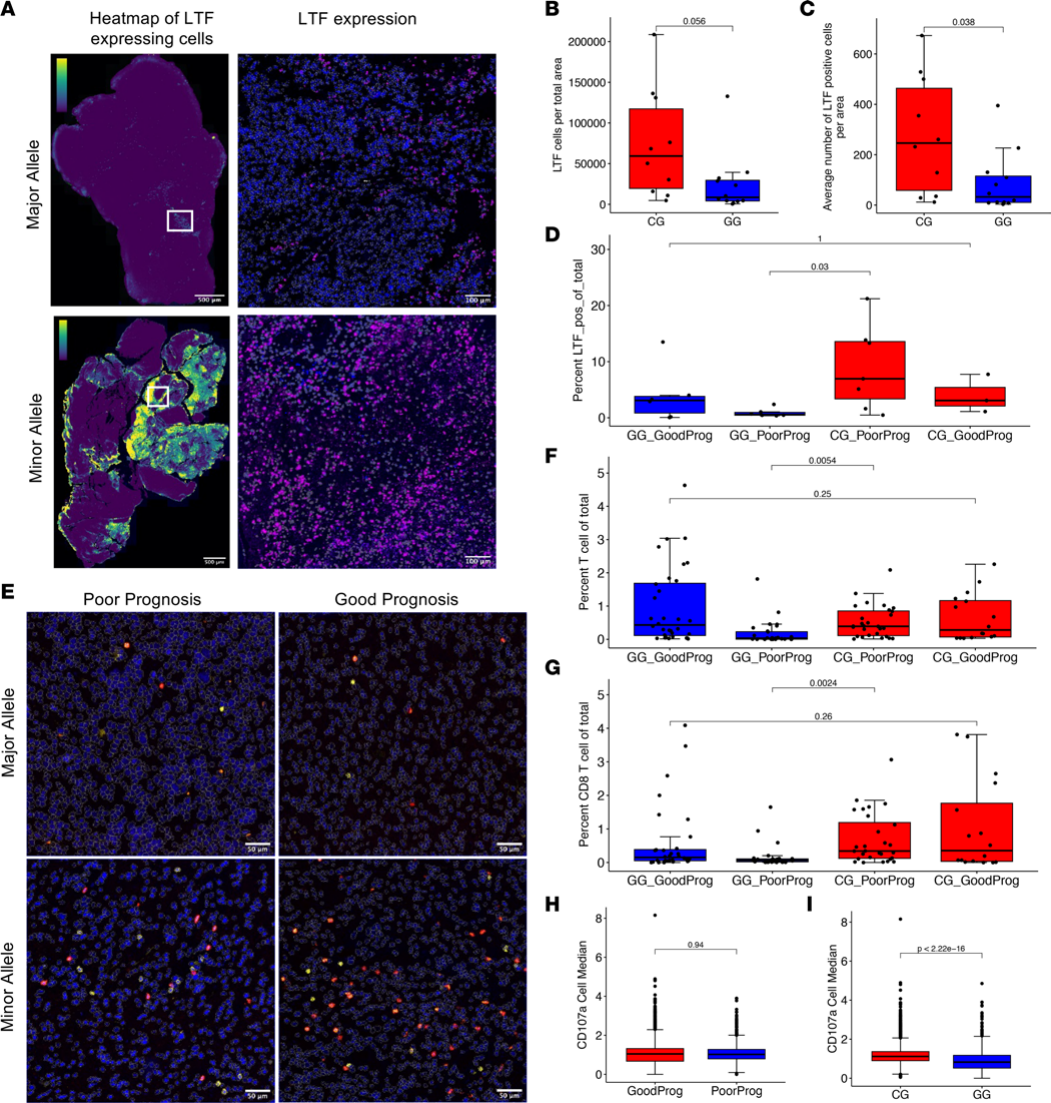
Immunofluorescence confirms enhanced T cell infiltration and CD8^+^ T cell activation in patients with GBM with the *MIF* SNP rs755622. (**A**) *LTF* expression was analyzed by immunofluorescence of matched tissue for *n* =22 samples from the RNA-Seq analysis to compare patients with the minor allele (C/G) (*n* = 10) and those with the major allele (G/G) (*n* = 12), with representative images of the data set shown as a whole view of the slide in the heatmap images (left column: yellow, increased density of staining). In a 20***×*** image of the slide, cells are outlined in white, with nuclei marked by DAPI and LTF pseudocolored purple. Left column: Full slide scans, zoomed to see the entire section. Scale bar: 500 µM. LTF is pseudocolored from purple to yellow scaled from 0 to 0.2. Right column: Representative zoomed in regions of major allele (top) and minor allele (bottom), where nuclei marked with blue DAPI and LTF colored purple. Scale bar: 100 µM. (**B**) The average LTF expression (mean fluorescence intensity) of cells per sample was compared between each genotype and the negative secondary-only control (*n* = 10 C/G and *n* = 12 G/G). (**C**) The quantity of LTF^+^ cells per total area was measured and then compared between the C/G and G/G genotypes using unpaired *t* test (*n* = 10 C/G and *n* = 12 G/G). (**D**) The percent of LTF^+^ cells of the total cells per sample was compared between each genotype group and further subdivided into prognostic categories based on greater than or less than median overall survival (poor prognosis contains *n* = 6 G/G and *n* = 7 C/G, good prognosis contains *n* = 6 G/G and *n* = 3 C/G). (**E**) Staining for CD3 and CD8 markers to determine CD8^+^ T cell infiltration, with representative images of each genotype and prognosis group (CD3, yellow; CD8, red; DAPI, blue). Scale bar: 100 µM. (**F** and **G**) Percent of T cells (**F**) and CD8^+^ T cells (**G**) of total cells per sample comparing the genotype/prognosis categories only. (**H**) CD107a expression of CD8^+^ T cells from all samples was compared between prognoses. (**I**) CD107a expression of CD8^+^ T cells was compared between the C/G genotype and G/G genotype.

To further interrogate the immune cell differences between patients with differing MIF genotypes, we utilized matched tissue from 22 of the patients from our RNA-Seq cohort (12 minor allele C/* and 10 major allele G/G). Staining for LTF confirmed the RNA-Seq analysis and showed increased LTF protein in minor allele patients (Figure 3, A–C). The percentage of LTF-expressing cells was further enhanced in minor allele patients with shorter survival spans, based on an overall survival of less than the median of the 34 samples in this cohort (Figure 3D). Additional assessment of T cell populations identified significant increases in total T cells in minor allele patients (Figure 3, E and F), and this increase appeared to mostly correspond to CD8^+^ T cells, based on similar results from quantifying total CD3^+^ or CD8^+^ cells (Figure 3, F and G). While analysis of the activation marker CD107a on CD8^+^ T cells showed no difference between patients with different prognoses (Figure 3H), CD107a was significantly increased in CD8^+^ T cells in patients with the minor allele (Figure 3I). These data support the conclusion that immune infiltration by a cytotoxic T cell population and LTF expression are increased in the tumor microenvironment of patients with the minor allele.

**Figure 4:**
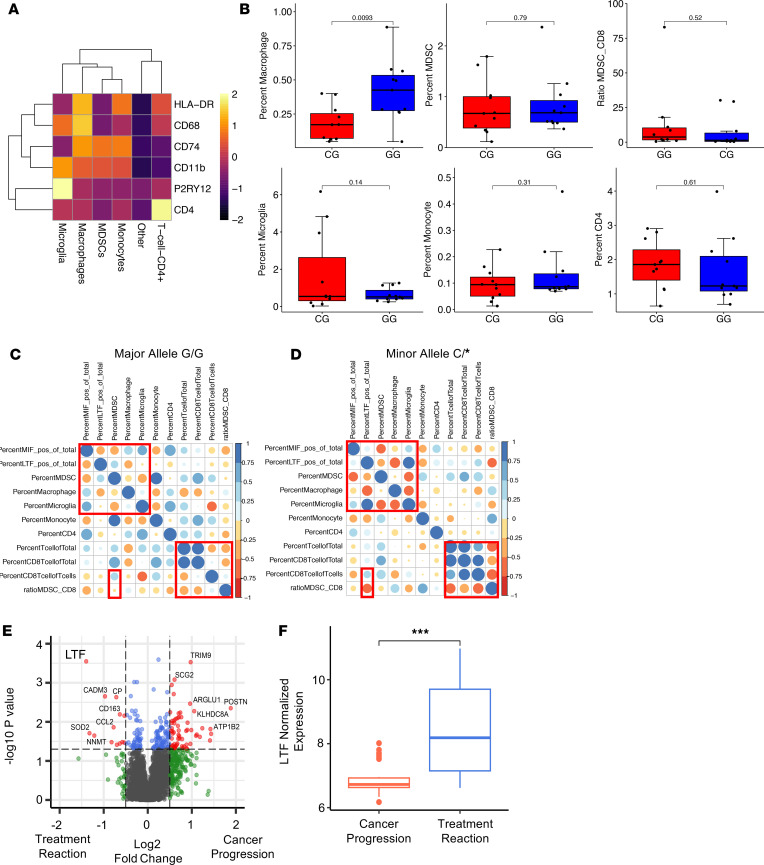
The MIF SNP rs755622 correlates with reduced macrophages and increased T cell infiltration by immunofluorescence. (**A**) A multiplex panel of myeloid antibodies was developed and included HLA-DR, CD68, CD74, CD11b, P2Ry12, and CD4, with the average expression shown for each cell type identified. (**B**) The percent of macrophages, MDSCs, microglia, monocytes, and CD4^+^ T cells and the ratio of MDSCs/CD8^+^ T cells were calculated as the percent of each cell type per sample out of the total cells identified, and *P* values were calculated using 2-tailed *t* test (*n* = 10 G/G and *n* = 12 C/G). (**C** and **D**) Correlation analyses were performed for immune cell infiltrates from each patient with the major allele genotype (*n* = 10 G/G) (**C**) and the minor allele genotype (*n* = 12) (**D**). Color scale and circle size are representative of the Pearson correlation coefficient. (**E**) NanoString spatial profiling comparing regions of *n* = 8 recurrent glioma samples where areas confirmed to have abnormal nuclear cellularity suggesting cancer were targeted. Comparing differential expression of these treatment response versus progressive regions highlighted increased *LTF* in treatment-reactive regions. (**F**) Of note, cases that ultimately presented clinically as treatment reactive had increased enrichment for *LTF* (***P < 0.001, *n* = 8).

While we observed differences in the lymphoid compartment of major and minor allele patients by RNA-Seq and validated these in patient samples, these initial assessments did not focus on specific myeloid cell subtypes that are known to be involved in GBM immune suppression (including microglia, monocytes, macrophages, and MDSCs). Again using matched samples from the patients included in the RNA-Seq study, we next stained for CD4^+^ T cells and these myeloid cell subtypes (*n* = 12 C/* and *n* = 10 G/G). First, we identified individual cell subtypes using the top quartile for lineage-specific markers (CD4^+^ T cells: CD4^+^CD11b^–^; macrophages: CD11b^+^CD68^+^HLA-DR^+^; microglia: P2RY12^+^; MDSCs: CD11b^+^CD74^+^CD68^–^HLA-DR^–^; monocytes: CD11b^+^HLA-DRA^+^P2RY12^–^CD68^–^) (Figure 4A). Heatmap representations of cell type markers are shown for all cells in the data set using the average cell MFI for each marker (Figure 4A). Representative images for each marker are shown, highlighting specific cell types from 1 sample section containing all cell types to demonstrate staining specificity for markers for the different cell types (Supplemental Figure 6A). Quantification revealed decreased macrophages in minor allele patients but no other major changes in MDSCs or the ratio of MDSCs to CD8^+^ T cells, microglia, monocytes, or CD4^+^ T cells between genotypes (Figure 4B). Due to the quantitative difficulties of assessing all myeloid cell lineages in an individual panel with lymphoid populations, we correlated cell types across samples per genotype (Figure 4, C and D). Using this approach, we found striking differences between genotypes, with the minor allele patients exhibiting an increase in CD8^+^ T cells and reductions in the myeloid compartment. Additionally, LTF positively correlated with microglia and negatively correlated with the ratio of MDSCs to CD8^+^ T cells. These data lead to the overall conclusion that minor allele patients have increased lymphocyte infiltration with reduced macrophage content and that LTF is associated with increased CD8^+^ T cells.

In an effort to better understand how the immune microenvironment plays a role in treatment response, we next developed a cohort of *n* = 8 recurrent glioma samples for spatial profiling using nanostring spatial genomics platform. In this study, we focused on areas confirmed to have abnormal nuclear cellularity, suggesting cancer cells were targeted and is responsive to treatment. We then compared differential expression of these treatment responsive regions versus progressive regions and found that immune-related genes, including *LTF*, were increased in treatment reactive regions (Figure 4E). Ultimately, cases that presented clinically as treatment reactive also had increased enrichment for *LTF* expression (Figure 4F).

### Patients with GBM with high LTF expression are immunologically activated.

**Figure 5:**
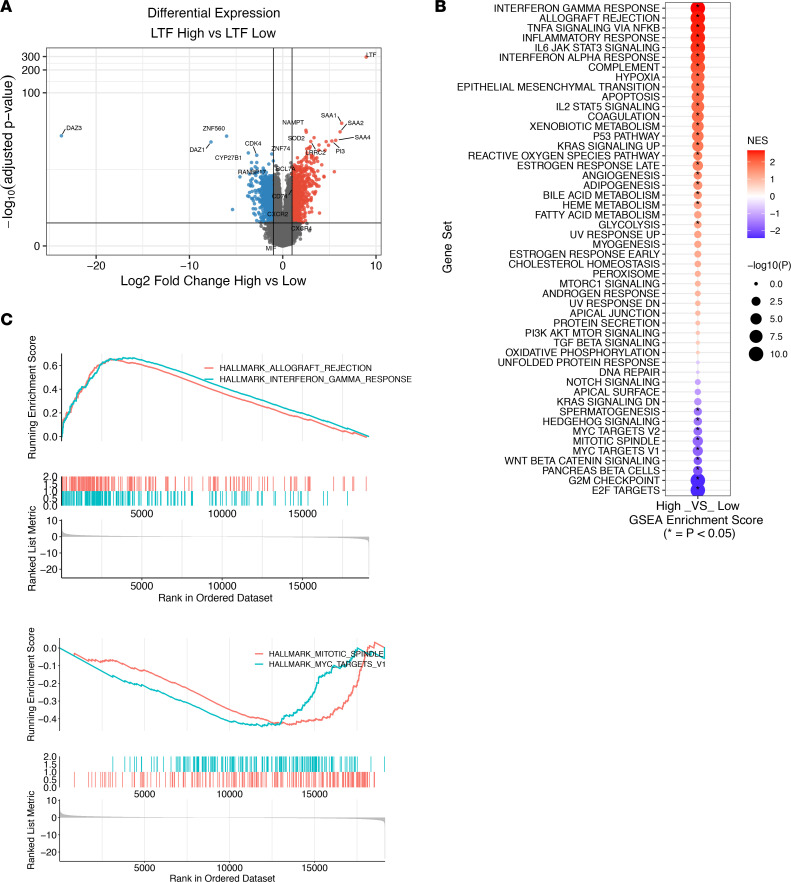
Patients with GBM with high *LTF* expression are immunologically activated. (**A**) TCGA_GBM mRNA-Seq data were analyzed to compare *LTF*-high (top 25% expression, *n* = 39) and *LTF*-low (bottom 25% expression, *n* = 39) patients, and differentially expressed genes are shown between the groups (red, > 1 log_2_FC and > –log_10_ adjusted *P* value; blue, < –1 log_2_FC and > –log_10_ adjusted *P* value). (**B**) GSEA was performed based on the ranked list of differentially expressed genes between *LTF*-high and *LTF*-low samples, with pathways enriched in *LTF*-high samples shown in red. Pathways highlighted in blue were enriched in *LTF*-low samples. (**C**) Highlighted GSEA plots of rank-ordered genes from the Hallmark pathways for Allograft Rejection and Complement Signaling, which are enriched in *LTF*-high patients, and Mitotic Spindle and Myc Targets, which are pathways that are enriched in *LTF*-low patients.

**Figure 6:**
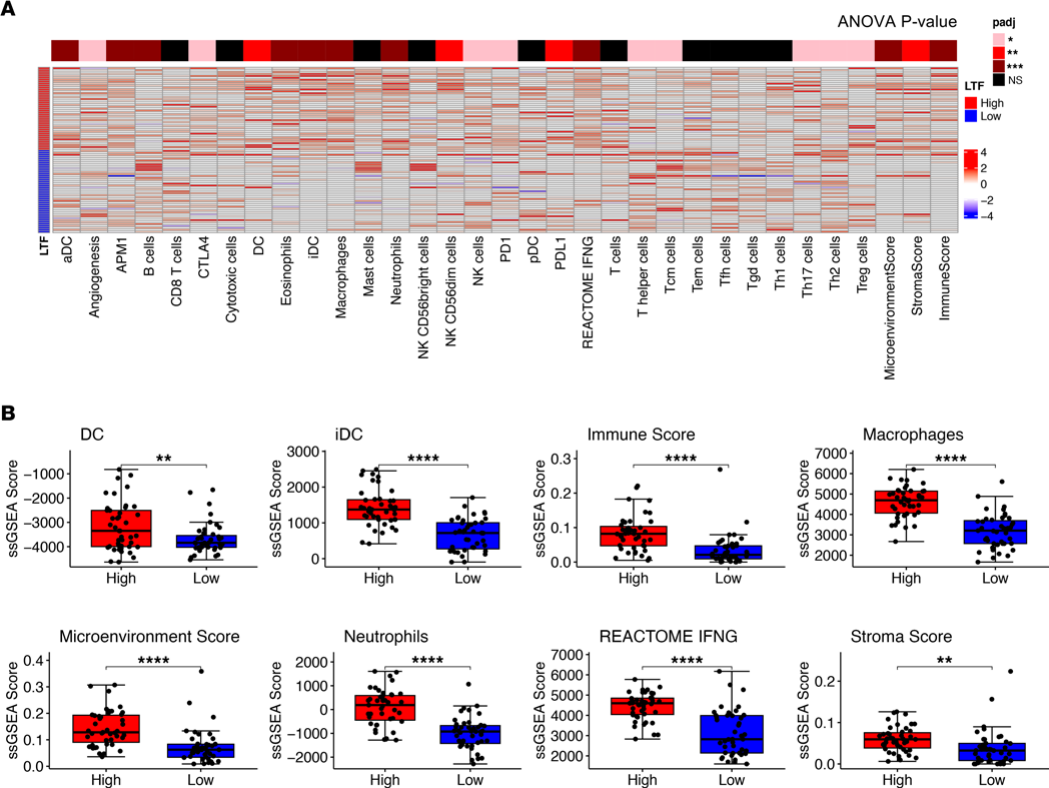
Patients with GBM with high *LTF* expression have increased immune infiltration. (**A**) Deconvolution analysis of the samples belonging the *LTF*-high (*n* = 39) and *LTF*-low (*n* = 39) groups showed increased immune cell type infiltration and increased immune scores, with ANOVA for multiple comparisons showing the *P* value by heatmap coloring. (**B**) Individual plots for significantly different estimated cell types and scores shown as individual box plots with unpaired *t* test (*n* = 39 samples per group).

Seeking to expand on these initial observations, we sought to identify the rs755622 SNP in The Cancer Genome Atlas (TCGA) data set. However, we were not able to detect the SNP in this data set because the marker is too far upstream to have read coverage in whole-exome sequencing data. Given the strong correlation between the *MIF* minor allele and *LTF* expression, we used *LTF* expression as a surrogate of *MIF* genotype and interrogated immune changes in the context of *LTF* expression. Notably, we found a similar differential expression profile between *LTF*-high (top 25%) and *LTF*-low (bottom 25%) patients in the TCGA compared with what we found between genotypes in our data set (Figure 5A). In agreement with our initial assessment, GSEA revealed an increase in immune activation pathways in the *LTF*-high patients, including Allograft Rejection and Complement Signaling, and a reduction in cancer-related pathways, including Mitotic Spindle and Myc Targets (Figure 5, B and C). To further identify cell type estimates between *LTF*-high and *LTF*-low samples, we performed deconvolution analyses and found increased immune and microenvironment scores, IFN-γ score, and macrophage content (Figure 6, A and B). However, we did not see significant differences between *LTF*-high and *LTF*-low samples in many T cell subsets, such as T central memory cells, T effector memory cells, T follicular helper cells, γδ T cells, and Th1 cells.

### rs2096525 serves as a surrogate for rs755622 to identify increased immune activation in GBM.

To determine whether these findings with respect to *LTF* gene signature recapitulate the rs755622 genotype, we examined possible associations with the rs2096525 SNP, which is in linkage disequilibrium with rs755622 but located within the first or second *MIF* intron. We interrogated the TCGA GBM whole-exome sequencing data set for the rs2096525 SNP and found the minor allele to be present in approximately 12% of patients with GBM (Supplemental Figure 7A). Differential expression analysis did not identify *LTF*, *MIF*, or the *MIF* receptors (Supplemental Figure 7B); however, functional GSEA did identify a similar increase in immune activation (Supplemental Figure 7, C and D). Additionally, a deconvolution analysis demonstrated similar increases in macrophages, neutrophils, and T cells for this SNP as observed by the rs755622 RNA-Seq study of Cleveland Clinic Foundation patients (Supplemental Figure 7E).

## Discussion

MIF, considered the first active cytokine discovered (37), has been extensively studied in the context of immune activation and the inflammatory response, as well as in tumor biology, where it has been shown to drive cancer cell proliferation and the generation of a tumor-promoting immune microenvironment (38). In GBM, functions of MIF include enhancement of CSC maintenance (39, 40), resistance to therapies including SOC chemotherapy temozolomide (41) and the antiangiogenic agent bevacizumab (42), and alteration of growth factor receptor signaling (34). However, the potential functional consequences of common *MIF* promoter polymorphisms, including the -173 SNP (rs755622), have not been well studied in the context of the inflammation associated with malignancies. Furthermore, the effects of *MIF* promoter polymorphisms have been studied even less often in CNS-associated disorders due to the strong expression quantitative trait locus (eQTL) in CNS tissues; however, we know that the immune cells play an integral role in CNS tumor progression and that they are in eQTL (Supplemental Figure 8). While the rs755622 SNP is associated with numerous inflammatory conditions and certain cancers, particularly those sensitive to immunotherapies (7, 38, 43, 44), we found no correlation with GBM incidence or prognosis in response to SOC therapy across 3 studied cross-institutional cohorts. This finding extended to the functional *MIF* promoter –794 CATT microsatellite (rs5844572) that is in linkage disequilibrium with the rs755622 SNP. We also observed no major difference in *MIF* gene expression level between genotypes, likely due to the elevated level of *MIF* in GBM or by the treatment of many patients with GBM with dexamethasone, which increases MIF production (45). However, we found evidence for distinct tumor immune microenvironments between SNP genotypes, with an increase in CD8^+^ T cells in the minor allele patients. This enhancement in immune response parameters correlated with an enhancement in *LTF* expression in the minor allele patients.

Our initial assessment of *MIF* genotypes revealed an association between the minor SNP allele and *LTF* that has not been previously described. In nonpathophysiological conditions, LTF is an iron-binding glycoprotein that functions to protect against pathogens and has been shown to have antiinflammatory activity. In cancer, LTF has been described to function in an antiproliferative manner. *LTF* expression is reduced in GBM compared with lower-grade brain tumors (46) and can inhibit GBM cell proliferation (47). LTF has also been studied as a nanoparticle carrier for a variety of preclinical cancer therapies, including in GBM, where it is able to penetrate the brain (48). In the context of MDSCs, we found that MIF enhances MDSC function in GBM and that LTF can induce MDSCs in pathological neonatal inflammatory conditions (49). While the MIF and LTF pathways have not been directly linked, our data suggest a likely interaction. *LTF* and *MIF* are likely not coregulated due to their location on separate chromosomes and their distinct transcription factors, but future studies interrogating the molecular relationship between LTF and MIF may provide additional insight into signaling networks that functionally link these 2 proteins in GBM.

The association of *LTF* with higher microenvironmental immune response and improved prognosis was further highlighted by our spatial profiling analysis. While both groups contained areas of cancer cellularity, treatment-reactive processes such as pseudoprogression have been associated with abundant global immune infiltration (50). Furthermore, patients experiencing reactions to treatment have better survival outcomes (51–53). Based on these results, it’s reasonable to hypothesize that *LTF* expression could be prognostic in the immunotherapeutic setting. Together, these data indicate a distinct activated immune environment in patients containing the minor allele of the *MIF* SNP that is associated with *LTF* expression and CD8^+^ T cell infiltration.

Numerous groups have reported on the association between CD8^+^ T cell infiltration and prognosis in GBM, with varying results; however, a majority of studies appear to show a positive association between T cell infiltration and overall survival (54–56). In more recent literature, a more nuanced point of view has emerged recognizing the specific impact of proliferating CD8^+^ T cells and/or the ratio of CD8^+^/CD4^+^ T cells on survival in patients with GBM (57, 58), supporting a necessary balance within the cellular immune response in GBM to improve current treatment strategies. The challenge of predicting outcomes with simple cell enumeration in a complex disease such as GBM is reflected in our study, as the *MIF* SNP was associated with increased CD8^+^ T cells, but not outcome, in our patient population. However, tumor-infiltrating lymphocytes (TILs), and CD8^+^ TILs in particular, are known to be critical for limiting tumor progression (57). In the context of immunotherapy, microenvironments high in CD8^+^ TILs are typically referred to as “hot” tumors and have been demonstrated to have a higher response rate to immunotherapies. While this phenotype is only seen in approximately 15% of GBM cases, this observation could indicate an immunotherapy-responsive subtype of GBM, which future studies should explore.

In analyzing the MIF SNP rs2096525, which is in linkage disequilibrium with rs755622, we were initially surprised to not identify overlap of top differentially expressed genes with our rs755622 RNA-Seq cohort. However, it is important to note that we utilized TruSeq methods of library preparation along with a deep-sequencing approach. Additionally, TCGA-GBM samples have many batch effects such as institution, sequencers, and potential necrotic samples that are difficult to control for in a comparison such as this. Together, these factors make it challenging to interpret the direct findings with this SNP from TCGA. However, the use of broader approaches such as GSEA did produce overlapping signatures similar to that of our cohort. This may indicate that these samples are overall more immune activated, but mechanistic studies of *LTF* expression in relation to the MIF SNP rs755622 and MIF itself are needed to understand whether *LTF* plays a direct role in immune activation.

Utilizing both RNA-Seq and matched tissue samples, we identified that MIF SNP minor allele patients with increased *LTF* expression also had an increase in CD8^+^ T cells and a reduction in macrophages, with no change in MDSCs. We also did not observe a consistent change in tumor-associated macrophages, and this could be due to a limitation of deconvolution methods in distinguishing myeloid subtypes. Taken together, this immune microenvironment may be more conducive to immune-activating strategies, and this should be assessed in future clinical trials. While our initial analysis revealed a high correlation between the *MIF* minor allele and elevated *LTF*, the association between the *MIF* SNP minor allele and *LTF* is indirect. This was due to the inability to efficiently identify *MIF* SNP status in large genomic data sets based on its location in the promoter region, which is not covered by whole-exome sequencing. Nonetheless, utilizing *LTF* at a median cut-off did not yield the same results as the top quartile of *LTF*, which more closely represents the frequency of the *MIF* SNP minor allele. Another limitation of our findings is that the *MIF* SNP has not been functionally characterized but is in linkage disequilibrium with the *MIF* –794 CATT repeat, which is associated with an increase in *MIF* production in immune cells and brain tissues.

The known genetic determinants of immunotherapy response in gliomas, including somatic mutations in *IDH*, are limited, and the present findings identify a common germline SNP linked to potential immunologic differences that may help inform clinical decision-making and be leveraged for the development of more effective immunotherapies.

## Methods

### DNA isolation and quantification.

Genomic DNA was extracted from the peripheral blood of patients with GBM using a Qiagen DNeasy Blood & Tissue Kit following the manufacturer’s protocols. DNA purity and concentration were measured using a Thermo Fisher Scientific NanoDrop spectrophotometer.

### SNP genotyping.

*MIF* SNP genotyping was performed using PCR amplification and subsequent restriction enzyme digestion with *AluI*. PCR was performed using Accuprime Pfx DNA polymerase (Thermo Fisher Scientific, catalog 12344-024) using 0.2 μmol forward primer (5′-CCCAAAGACAGGAGGTAC-3′) and 0.2 μmol reverse primer (5′-ATGATGGCAGAAGGACCAG-3′). PCR was run as follows: 95°C for 5 minutes, followed by 35 cycles of 95°C for 30 seconds, 60°C for 45 seconds, and 68°C for 1 minute. Following the 35 cycles, there was a final 68°C elongation step for 5 minutes, followed by storage at 4°C. After amplification, the PCR product was confirmed on a 1% agarose gel by identification of an approximately 500 bp product. After confirmation, 10 μL of PCR product was mixed with 2 μL 10***×*** CutSmart buffer, 1 μL AluI, and 7 μL water and digested at 37°C for 1 hour. After digestion, the alleles containing a G (non SNP) produced a 450 bp fragment, while the alleles with a C (rs755622) produced a 270 bp fragment.

### SNP calling.

Raw BAM files from the TCGA_GBM cohort were utilized to analyze the rs2096525 *MIF* SNP from whole-exome sequencing data aligned by the TCGA. The SNP rs2096525 genotype was identified via use of HaplotypeCaller, where samples with alternative counts at the reference position chr22: 23894632 were identified. After classification of the samples by genotype, the phenotype data were downloaded via TCGA, and survival analysis was performed using log-rank test via R version 4.1.0.

### RNA-Seq.

Flash-frozen tissue was obtained from the Rose Ella Burkhardt Brain Tumor Center at the Cleveland Clinic under IRB 2559 and corresponded to *n* = 34 patients with previously identified rs755622 SNP status from matched WBC pellets (*n* = 17 C/*, and *n* = 17 G/G). Within each group, patients were selected who had undergone full Stupp protocol SOC treatment and were evenly divided by sex and prognosis (<6 months PFS or >6 months PFS). Samples were processed and sequenced by Genewiz. Briefly, RNA was extracted by Qiagen RNeasy kit, and then the library was prepared using TruSeq library preparation. Average sequencing depth was 40 Mbp per sample.

FASTQ files were aligned to the hg19 using STAR aligner with default parameters. Fragments were counted using Rsamtools with UCSC.hg19.knownGene transcript database. Raw counts were used in DESeq2 downstream for differential expression comparing the rs75662 SNP status groups. All differential expression results are provided in Supplemental Data File 1.

### TCGA RNA-Seq data.

Processed count data from TCGA_GBM mRNA data set were downloaded from the Broad Firehose (https://gdac.broadinstitute.org/runs/stddata__2016_01_28/data/GBM/20160128/gdac.broadinstitute.org_GBM.mRNAseq_Preprocess.Level_3.2016012800.0.0.tar.gz), where the raw count file GBM.uncv2.mRNAseq_raw_counts.txt was utilized for downstream analysis.

### Differential expression.

Raw counts from TCGA_GBM were analyzed using the R package DESeq2 version 1.29 in R version 4.0.1. After identification of the germline rs2096525 SNP status, the patient samples containing the minor allele were compared with patients homozygous for the major allele for differential expression.

### Pathway enrichment.

GSEA was performed based on the ranked list of differentially expressed genes by log fold-change value using the R package Gene Set Variation Analysis (GSVA). GSEA results was potted using the enrich plot R package. Hallmark gene sets used for GSEA downloaded from gsea-msigdb.

### ssGSEA.

The nCounter PanCancer Immune Profiling Panel gene set was used for immune infiltration deconvolution signatures. Each signature was used with ssGSEA using GSVA R package version 1.40.1. Microenvironment score, stromal score, and immune score were generated using xCell R package version 1.1.0 (59). Comparing signature scores between groups was performed using 2-way ANOVA with the *P* values shown for each comparison above the deconvolution heatmaps. All deconvolution and *P* value heatmaps were generated using pheatmap version 1.0.12.

### Immunofluorescence staining.

Serial sections (7 μm thick) from each sample (formalin-fixed paraffin-embedded tumor biopsies) were stained with 3 different sets of markers and indicated below:

Set 1 (triple immunofluorescence staining): DAPI, CD3 (ab11089, Abcam, 1:50), CD8 (85336, Cell Signaling, 1:50), CD107a (NBP2-52721, Novus Bio, 1:50); Set 2 (double immunofluorescence staining): DAPI, MIF (MAB2892, R&D systems, 1:500), LTF (HPA059976, Atlas, 1:100); and Set 3 (multiplex staining): DAPI, CD74 (ab1794, Abcam, 1:200), CD11b (ab133357, Abcam, 1:200), P2RY12 (NBP2-33870, Novus Bio, 1:200), HLA-DR (ab20181, Abcam, 1:200), CD68 (790-2931, Ventana, 1:200), CD4 (ab133616, Abcam, 1:200).

For staining, slides were baked at 60°C prior to deparrifinization. Slides were then deparaffinized using a Leica Autostaine XL, and antigen retrieval was performed using a sodium citrate buffer (pH 6) with slides steamed in a pressure cooker at 110°C for 10 minutes. Slides were then cooled to room temperature and transferred to water for 5 minutes prior to TBST for 15 minutes. Primary antibody was placed at the above concentrations and incubated in a humid chamber overnight at 4°C. Slides were placed on a Biocare Intellipath Staining platform for blocking (3% donkey serum), secondary antibody incubations, and Hoechst/DAPI staining (Thermo Fisher Scientific, H1399, 1 mg/mL). The following secondary antibodies were used: rabbit Cy3 (Jackson ImmunoResearch, 711-165-152, 1:250), mouse 488 (Jackson ImmunoResearch, 715-545-151, 1:250), rat Cy3 (Jackson ImmunoResearch, 712-165-153, 1:250), and rabbit Cy5 (Jackson ImmunoResearch, 711-175-152, 1:250). Slides were manually cover-slipped with an aqueous mounting medium.

### Imaging.

Whole-tissue sections were imaged with the multispectral capabilities of the Vectra Polaris Automated Quantitative Pathology Imaging System (Akoya Bioscience Inc.). Multispectral images were then unmixed in inForm (Akoya Biosciences Inc., version 2.5) to obtain component images for each individual marker and tissue autofluorescence. Component image tiles were stitched and saved as OME-TIFF (Open Microscopy Environment) format for analysis, storage, and archival.

### Image analysis.

The open-source image analysis software QuPath was used for the detection and classification of cells. For each slide, 10–20 regions of interest (ROI) were selected to represent different areas of the whole section while avoiding imaging, staining, and sectioning artifacts. StarDist (60), a deep-learning algorithm, along with a pretrained model was used within QuPath for detecting cell nuclei from the DAPI channel. For each cell, intensity measurements were used to determine its positivity for each marker in the panel. A custom script with a manual decision tree (61) was implemented in QuPath to classify cells based on their positivity. Representative images were extracted using QuPath, and single-cell data for each sample were exported as.csv files for further analysis and charting in R version 4.0.1.

### NanoString GeoMx.

Eight deidentified GBM samples taken from second surgeries for potentially novel MRI enhancement were evaluated using a NanoString GeoMx Digital Spatial Profiler using NanoString Human Whole Transcriptome Atlas probes. Samples were stained with Novus Sox10–Alexa Fluor 647-conjugated (catalog NBP2-59621AF647), Santa Cruz CD68–Alexa Fluor 594-conjugated (catalog sc-20060 AF594), and NanoString Syto13-conjugated (catalog S7575) antibodies to select for areas of cancer cellularity. This was confirmed with overlayed clinical IHC imaging for OLIG2, KI67, and P53. Normalization and differential expression analysis were performed using NanoString GeoMx software following the default manufacturer protocols (62–65). Clinical diagnosis of lesions was noted prior to deidentification, with 4 samples corresponding to treatment reaction and 4 samples to cancer progression.

### Statistics.

Demographic and clinical characteristics were evaluated between clinical cohorts. Two-way ANOVA and χ^2^ tests were performed to assess differences in continuous and categorical variables, respectively. Additionally, demographic and clinical characteristics were assessed for association with the *MIF* SNP rs755622. For this assessment, 2-tailed *t* tests were performed to assess differences in continuous data. All statistics were generated in R version 4.0.1.

Overall and PFS of each clinical cohort was assessed for *MIF* SNP rs755622. Kaplan-Meier (KM) analysis was performed to evaluate the difference in survival and recurrence between patients with a GG genotype and with CC or CG genotypes. These analyses were also performed among only those patients who had received SOC. Log-rank tests were performed to assess differences in KM curves. Univariate and multivariable Cox proportional hazards models were generated to assess the effect of *MIF* SNP rs755622 on overall and PFS. The proportional hazards assumption was assessed and not found in violation. Multivariable models were adjusted for age, sex, surgery, and SOC. Hazard ratios (HR) and 95% CI are reported. All statistics were generated in R version 4.0.1.

### Study approval.

For Cleveland Clinic, peripheral blood samples from 451 patients with GBM were collected through the Rose Ella Burkhardt Brain Tumor and Neuro-Oncology Center under approved IRB protocol no. 2559. WBCs from each blood sample were isolated via Ficoll gradient and then snap frozen and stored at –80°C for research use. For this study, we selected all available GBM samples. For Moffitt Cancer Center, salivary DNA samples collected using Oragene kits were available for 386 recently diagnosed patients with GBM under IRB protocol no. MCC 15004. DNA was extracted and stored in aliquot pellets at –80°C for future research use. For Case Western Reserve University/University Hospitals of Cleveland, peripheral blood samples from 131 patients with GBM were collected through the Ohio Brain Tumor Study at Case Western Reserve University, under approval from University Hospitals IRB CC296. Clinical and pathological data were gathered for each patient. Patient blood samples were collected and processed at the time of consent.

### Data availability.

The RNA-Seq data generated and analyzed during the current study are available in the Gene Expression Omnibus (GEO) repository under accession no. GSE232434. The data can be accessed at: https://protect-us.mimecast.com/s/i2OmCmZ6rWHprGyBlCNZghk?domain=ncbi.nlm.nih.gov The processed data, including normalized counts and differential gene expression analyses, are also available in the supplementary materials of this manuscript (Supplemental Data File 1).

Differential expression outputs are also provided, along with summarized imaging data in the Supplemental Data File 1. All code used to generate figures can be requested from the first author TA.

## Author contributions

TJA, MMG, BO, MAV, RB, TAC, MSA, and JDL conceived the experiments; TJA, MMG, BO, DB, AZ, VM, KT, MM, AR, AL, CN, GR, KAW, GC, NP, TTT, KM, and AS performed experiments and analysis; CMD, JMB, KME, CMH, JSBS, MAV, RB, TAC, MSA, and JDL supervised and participated in analysis; MAV, RB, TAC, MSA, and JDL supervised the project and provided financial support; and TJA, RB, TAC, MSA, and JDL wrote the manuscript. WW conceived experiments and performed experiments and analysis. All authors had final approval of the manuscript.

## Supplementary Material

Supplemental data

Supporting data values

## Version 1 05/30/2023

In-Press Preview

## Version 2 07/10/2023

Electronic publication
