# Supplementary material for: The MIF promoter SNP rs755622 is associated with immune activation in glioblastoma
Source: JCI Insight. 2023 Jul 10;8(13):e160024. doi: 10.1172/jci.insight.160024 (PMC10371339; doi:10.1172/jci.insight.160024)
Supplement: Supplemental data [file jciinsight-8-160024-s229.pdf]

Supplemental Table 1- MIF snp rs755622 highlighted literature in inflammatory associated conditions

PMID	Title
PMID: 19941661	A MIF haplotype is associated with the outcome of patients with severe sepsis: a case control study
PMID: 20388640	Predictors of response to intra-articular steroid injection in psoriatic arthritis
PMID: 20447688	The MIF -173G/C polymorphism and risk of childhood acute lymphoblastic leukemia in a Chinese population
PMID: 16380915	Replication of putative candidate-gene associations with rheumatoid arthritis in >4,000 samples from North America and Sweden: association of susceptibility with PTPN22, CTLA4, and PADI4
PMID: 17585860	Macrophage migration inhibitory factor in acute lung injury: expression, biomarker, and associations.
PMID: 20811626	Genetic variants in inflammation-related genes are associated with radiation-induced toxicity following treatment for non-small cell lung cancer.
PMID: 23402792	Macrophage migration inhibitory factor (MIF): genetic evidence for participation in early onset and early stage rheumatoid arthritis
PMID: 24151396	Macrophage migration inhibitory factor gene polymorphisms in inflammatory bowel disease: An association study in New Zealand Caucasians and meta-analysis
PMID: 26107210	Contribution of Macrophage Migration Inhibitory Factor -173G/C Gene Polymorphism to the Risk of Cancer in Chinese Population
PMID: 26542751	Functional polymorphisms in the gene encoding macrophage migration inhibitory factor (MIF) are associated with active pulmonary tuberculosis
PMID: 29545822	Macrophage Migration Inhibitory Factor -173 G/C Polymorphism: A Global Meta-Analysis across the Disease Spectrum.
PMID: 32560699	MIF -173G/C (rs755622) polymorphism modulates coronary artery disease risk: evidence from a systematic meta-analysis.
PMID: 22113576	Polymorphisms in immune function genes and non-Hodgkin lymphoma survival.
PMID: 24530749	Macrophage migration inhibitory factor: association of -794 CATT5-8 and -173 G>C polymorphisms with TNF-α in systemic lupus erythematosus
PMID: 27696094	A Macrophage Migration Inhibitory Factor Polymorphism Is Associated with Autoimmune Hepatitis Severity in US and Japanese Patients
PMID: 29661540	MIF functional polymorphisms (-794 CATT 5-8 and -173 G>C) are associated with MIF serum levels, severity and progression in male multiple sclerosis from western Mexican population
PMID: 29996006	MIF-173G/C (rs755622) polymorphism as a risk factor for acute lymphoblastic leukemia development in children
PMID: 30747392	Macrophage migration inhibitory factor polymorphisms are a potential susceptibility marker in systemic sclerosis from southern Mexican population: association with MIF mRNA expression and cytokine profile
PMID: 31978276	Association of the genetic variants (-794 CATT5-8 and -173 G > C) of macrophage migration inhibitory factor (MIF) with higher soluble levels of MIF and TNFα in women with breast cancer

Supplemental Table 1. *MIF* SNP rs755622 highlighted literature in inflammatory-associated conditions. Summary highlighting selected articles pertaining to rs755622 history and research in inflammatory conditions to provide the relative clinical importance of this germline SNP.

Supplemental Table 2- Clinical Cohort summary for samples used in RNA-sequencing study

**Characteristic**	**CC**, N = 1[1]	**CG**, N = 16[1]	**GG**, N = 17[1]
race			
black	0 (0%)	1 (6.2%)	1 (5.9%)
non hispanic white	1 (100%)	15 (94%)	16 (94%)
age	59 (59, 59)	64 (53, 69)	59 (49, 66)
sex	1 (100%)	6 (38%)	6 (35%)
kps.at.diagnosis			
60	0 (0%)	1 (6.2%)	0 (0%)
70	0 (0%)	1 (6.2%)	1 (5.9%)
80	0 (0%)	8 (50%)	4 (24%)
90	1 (100%)	6 (38%)	12 (71%)
deletion_1p	0 (0%)	1 (6.2%)	2 (12%)
deletion_19q	0 (0%)	5 (31%)	1 (6.2%)
idh1_mutated	0 (0%)	0 (0%)	1 (5.9%)
mgmt_methylated	0 (NA%)	2 (33%)	4 (80%)
ki_67	5 (5, 5)	25 (19, 32)	25 (14, 32)
egfr_amplified	0 (0%)	9 (56%)	5 (31%)
pfs	19 (19, 19)	8 (5, 21)	25 (5, 51)
os	27 (27, 27)	12 (10, 25)	36 (9, 82)

[1]n (%); Median (IQR)

Supplemental Table 2. Clinical cohort summary for samples used for RNA-sequencing. Summary of the patients used for RNAseq analysis from the CCF cohort (n=17 G/* and n=17 G/G patients). Molecular markers and clinical data available were selected along with overall survival and sex to evenly distribute similar cohorts of patients and minimize confounder.

Supplemental Figure 1- Multivariate and univariate analysis of the MIF SNP rs755622 in GBM cohorts

A

Table 1: Patient Characteristics by dataset

	Level	Overall N = 966	Case N = 131	Cleveland Clinic N = 449	Moffitt N = 386	p
Age at diagnosis						
mean (SD)		60.45 (12.87)	62.00 (11.13)	60.78 (13.30)	59.54 (12.88)	0.129[a]
median [IQR]		61.07 [53.00, 69.00]	64.00 [55.00, 69.00]	61.41 [53.29, 70.22]	61.00 [52.00, 69.00]	0.239[b]
Sex (%)	Female	344 (35.6)	47 (35.9)	150 (33.4)	147 (38.1)	0.371[c]
	Male	622 (64.4)	84 (64.1)	299 (66.6)	239 (61.9)	
Surgery status (%)	Gross total	358 (37.1)	75 (57.3)	156 (34.7)	127 (32.9)	<0.001[c]
	Subtotal	328 (34.0)	53 (40.5)	163 (36.3)	112 (29.0)	
	Other	280 (29.0)	3 (2.3)	130 (29.0)	147 (38.1)	
Standard of care (%)*	Yes	816 (84.5)	78 (59.5)	384 (85.5)	354 (91.7)	<0.001[c]
	No	142 (14.7)	45 (34.4)	65 (14.5)	32 (8.3)	
	unknown	8 (0.8)	8 (6.1)	0 (0.0)	0 (0.0)	
KPS (%)	<=70	206 (21.3)	56 (42.7)	65 (14.5)	85 (22.0)	<0.001[c]
	70 - 80	265 (27.4)	16 (12.2)	142 (31.6)	107 (27.7)	
	80 - 90	389 (40.3)	21 (16.0)	228 (50.8)	140 (36.3)	
	90 - 100	38 (3.9)	1 (0.8)	7 (1.6)	30 (7.8)	
	Not Available	68 (7.0)	37 (28.2)	7 (1.6)	24 (6.2)	
SNP Status	CC or CG	324 (33.5)	42 (32.1)	146 (32.5)	136 (35.2)	0.658[c]
	GG	642 (66.5)	89 (67.9)	303 (67.5)	250 (64.8)	
Overall Survival in months (median [IQR])		13.84 [7.38, 23.20]	12.40 [5.49, 20.75]	13.77 [7.00, 26.13]	14.54 [8.95, 21.96]	0.024[b]
Vital Status (%)	Alive	75 (7.8)	3 (2.3)	44 (9.8)	28 (7.3)	0.016[c]
	Deceased	891 (92.2)	128 (97.7)	405 (90.2)	358 (92.7)	
Recurrence free Survival in months (median [IQR])		6.87 [3.32, 12.45]	6.16 [3.95, 12.42]	5.57 [2.83, 12.23]	8.20 [5.23, 13.03]	<0.001[b]
Recurrence Status	Yes	629 (65.1)	74 (56.5)	330 (73.5)	225 (58.3)	
	No	173 (17.9)	56 (42.7)	117 (26.1)	0 (0.0)	<0.001[c]
	Unknown	164 (17.0)	1 (0.8)	2 (0.4)	161 (41.7)	

a.One-way analysis of Variance.
b.Kruskal-Wallis Test
c.Chi-square or Fisher's exact test as appropriate.
*Standard of Care includes surgery with a full 6 weeks of Radiation and Temodar.
31 patients with missing information (7 – SNP status, 23 – Overall survival, 2 – vital status) were excluded from the analysis.

B

Table 2: Patient Characteristics by genotype (all data combined)

	Level	Overall N = 966	CC or CG N = 324	GG N = 642	P-value
Age at diagnosis					
mean (SD)		60.45 (12.87)	60.93 (12.48)	60.21 (13.07)	0.406[a]
median [IQR]		61.07 [53.00, 69.00]	62.00 [53.00, 69.53]	61.00 [53.00, 69.00]	0.441[b]
Sex (%)	Female	344 (35.6)	135 (41.7)	209 (32.6)	0.007[c]
	Male	622 (64.4)	189 (58.3)	433 (67.4)	
Surgery status (%)	Gross total	358 (37.1)	115 (35.5)	243 (37.9)	0.514[c]
	Subtotal	328 (34.0)	118 (36.4)	210 (32.7)	
	Other	280 (29.0)	91 (28.1)	189 (29.4)	
Standard of care (%)*	Yes	816 (84.5)	258 (79.6)	558 (86.9)	0.012[c]
	No	142 (14.7)	63 (19.4)	79 (12.3)	
	unknown	8 (0.8)	3 (0.9)	5 (0.8)	
KPS (%)	<=70	206 (21.3)	65 (20.1)	141 (22.0)	0.900[c]
	70 - 80	265 (27.4)	89 (27.5)	176 (27.4)	
	80 - 90	389 (40.3)	131 (40.4)	258 (40.2)	
	90 - 100	38 (3.9)	15 (4.6)	23 (3.6)	
	Not Available	68 (7.0)	24 (7.4)	44 (6.9)	
Overall Survival in months (median [IQR])		13.84 [7.38, 23.20]	13.26 [7.07, 21.73]	14.13 [7.62, 24.24]	0.345[b]
Vital Status (%)	Alive	75 (7.8)	26 (8.0)	49 (7.6)	0.93[c]
	Deceased	891 (92.2)	298 (92.0)	593 (92.4)	
Recurrence free Survival in months (median [IQR])		6.87 [3.32, 12.45]	6.77 [3.41, 11.13]	7.00 [3.30, 13.04]	0.247[b]
Recurrence Status	Yes	629 (65.1)	200 (61.7)	429 (66.8)	
	No	173 (17.9)	66 (20.4)	107 (16.7)	0.253[c]
	Unknown	164 (17.0)	58 (17.9)	106 (16.5)	

a.Independent t test.
b.Mann-Whiteney U test
c.Chi-square or Fisher's exact test as appropriate.
*Standard of Care includes surgery with a full 6 weeks of Radiation and Temodar.
31 patients with missing information (7 – SNP status, 23 – Overall survival, 2 – vital status) were excluded from the analysis.

C

Single Nucleotide Polymorphism	Allele Frequency Controls (%) 1000 Genomes European, n=1,006	Allele Frequency GBM patients(%) CCF, n=449	Allele Frequency GBM patients (%) Moffitt, n=386	Allele frequency GBM patients (%) Case Western, n=131
Major Allele (G)	81.9	82.6	80.9	82.4
Minor Allele (C)	18.6	17.4	19.0	17.6

D

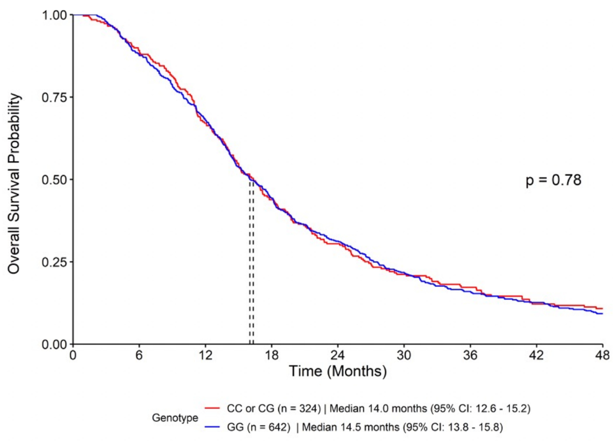

E

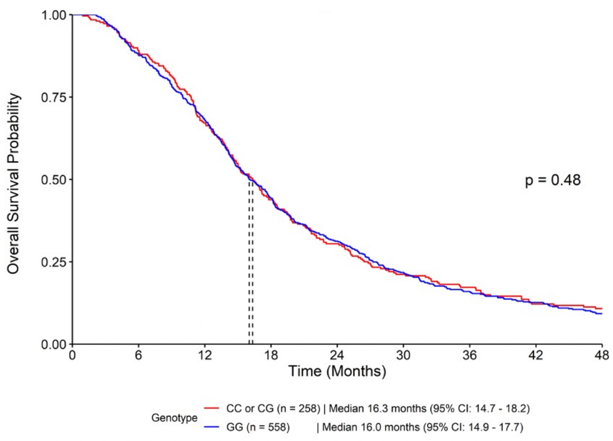

Table 3: Univariate and multivariable Cox proportional hazards models predicting overall and progression-free survival for MIF SNP rs755622 in GBM cohorts

	Overall Survival				Progression-Free Survival			
	Univariate		Multivariable*		Univariate		Multivariable*	
	Hazard Ratio (95% CI)	P	Hazard Ratio (95% CI)	P	Hazard Ratio (95% CI)	P	Hazard Ratio (95% CI)	P
Genotype								
GG	ref	--	ref	--	ref	--	ref	--
CC or CG	1.02 (0.89-1.17)	0.781	1.07 (0.81-1.08)	0.373	1.02 (0.86-1.21)	0.787	1.06 (0.90-1.26)	0.485

*Adjusted for age, sex, surgery, and standard of care

Supplemental Figure 1. Univariate and multivariable analysis of the *MIF* SNP rs755622 in GBM cohorts. Germline DNA was acquired from PBMCs or saliva samples from n=449 (Cleveland Clinic), n=386 (Moffitt Cancer Center), and n=131 (Case Western) GBM patients and then tested for the MIF SNP rs755622 using PCR with restriction enzyme digestion as described in detail in the methods. (**A**) Descriptive statistics of clinical features of GBM including sex, surgery type, standard of care, KPS, and SNP status including all cohorts of GBM patients. Primary features known to associate with outcome such as higher KPS, total as compared to subtotal tumor resection and treatment with standard of care all confer a survival advantage. (**B**) Descriptive statistics combining data from all three cohorts of GBM patients demonstrated statistically significant differences in allele frequencies of the *MIF* SNP rs755622 (minor 'C' allele containing genotypes vs the homozygous major 'G' allele genotype) for standard-of-care treatment and sex status. (**C**) The allele frequencies for *MIF* rs755622 are similar in the three GBM cohorts and the 1000 Genomes European cohort using Hardy-Weinberg principle and chi-square test for differences from the reference control cohort. (**D**) Univariate analysis of overall survival across all cohorts shows no survival difference by log-rank test. (**E**). Restricting to patients uniformly treated with standard of care, we observed no significant difference in overall survival according to *MIF* rs755622 genotype (CC/CG versus GG) by the log-rank test. (**F**). Univariate and multivariable Cox proportional hazards models predicting overall and progression-free survival for *MIF* SNP rs755622 in GBM cohorts.

Supplemental Figure 2- MIF CATT repeat analysis in GBM patients shows correlation with rs755622

A

Genotype	Patient #	Percent GBM Patients	Percent Controls
5/5	37	7.1	8.2
5/6	147	28.4	29.0
5/7	31	6.0	8.9
5/8	1	0.1	0.0
6/6	212	40.9	36.6
6/7	78	15.6	15.7
6/8	2	0.1	0.0
7/7	10	1.9	0.5
7/8	0	0.0	1.1
5 containing	184	35.5	37.2
7 containing	23	23.6	26.2

B

Segregation Status	Number of Patient Alleles	Percent
Co-segregation of G SNP with 5/6 CATT repeats	844	94.2
Separate segregation of G SNP with 5/6 CATT repeats	52	5.8
Co-segregation of C SNP with 7/8 CATT repeats	127	96.2
Separate segregation of C SNP with 7/8 CATT repeats	5	3.8

Supplemental Figure 2. *MIF* CATT repeat analysis in GBM patients shows correlation with rs755622. (**A**) The CATT microsatellite in the *MIF* promoter upstream of the *MIF* SNP rs755622 was analyzed by capillary electrophoresis in the Cleveland Clinic cohort. (**B**) Those patients with both rs755622 SNP information and CATT repeat information were correlated to confirm linkage disequilibrium, which showed approximately 95% segregation of the 5/6 repeat and the major allele at rs755622, while 7/8 CATT repeats were linked with the minor allele of rs755622.

Supplemental Figure 3- Univariate analysis of clinical cohorts for MIF snp rs755622 (Overall Survival)

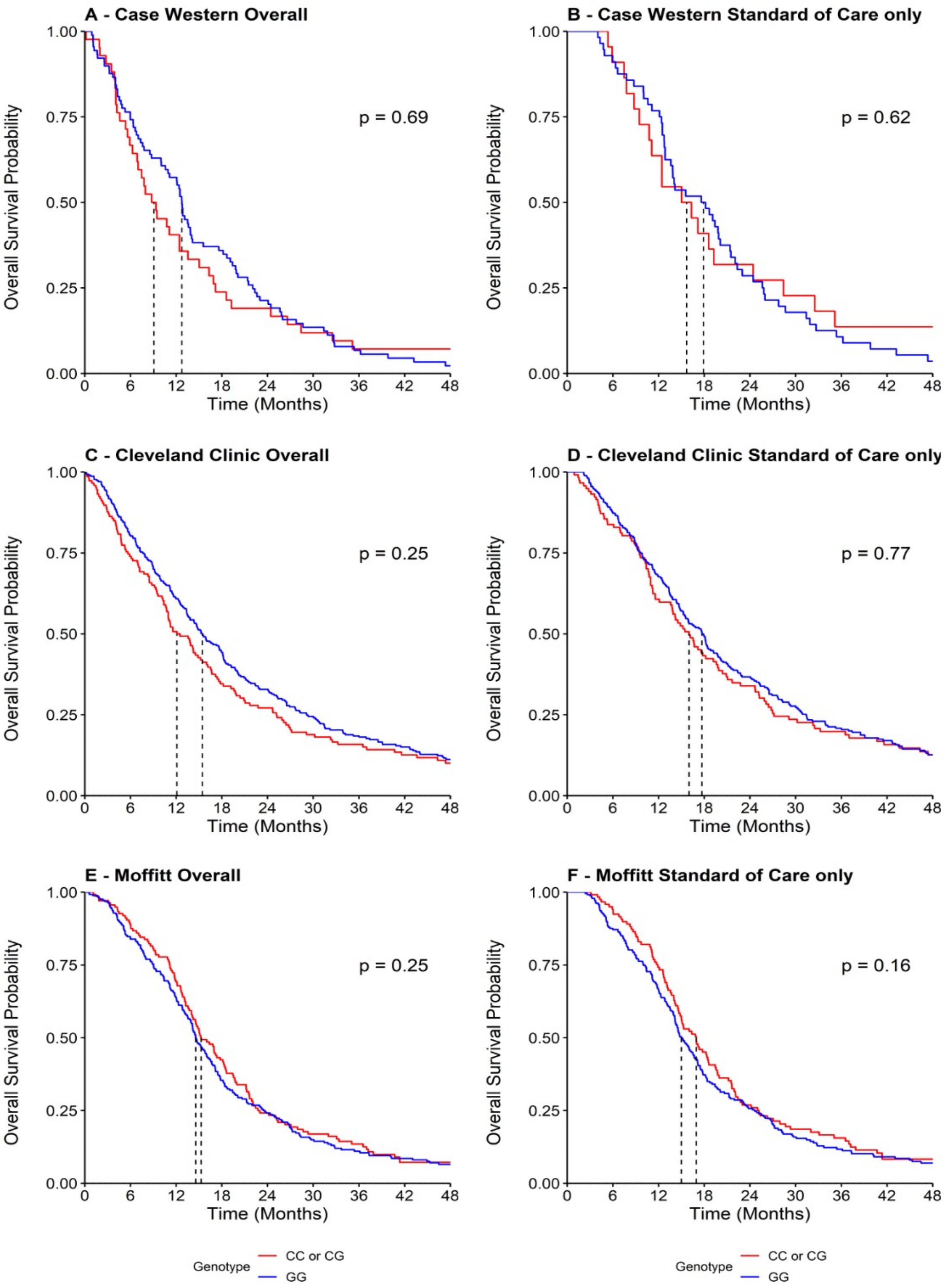

*Vertical lines indicate median survival time

Supplemental Figure 3. Univariate analysis of clinical cohorts for *MIF* SNP rs755622 (overall survival). (**A-F**) Univariate analysis of overall survival comparing patients with the minor allele to patients homozygous for the major allele across all samples (left column) and then those who received full standard-of-care treatment protocol (right column), with log rank p-value comparing each group provided to the right of each curve.

Supplemental Figure 4- Univariate analysis of clinical cohorts for MIF snp rs755622 (Progression Free Survival)

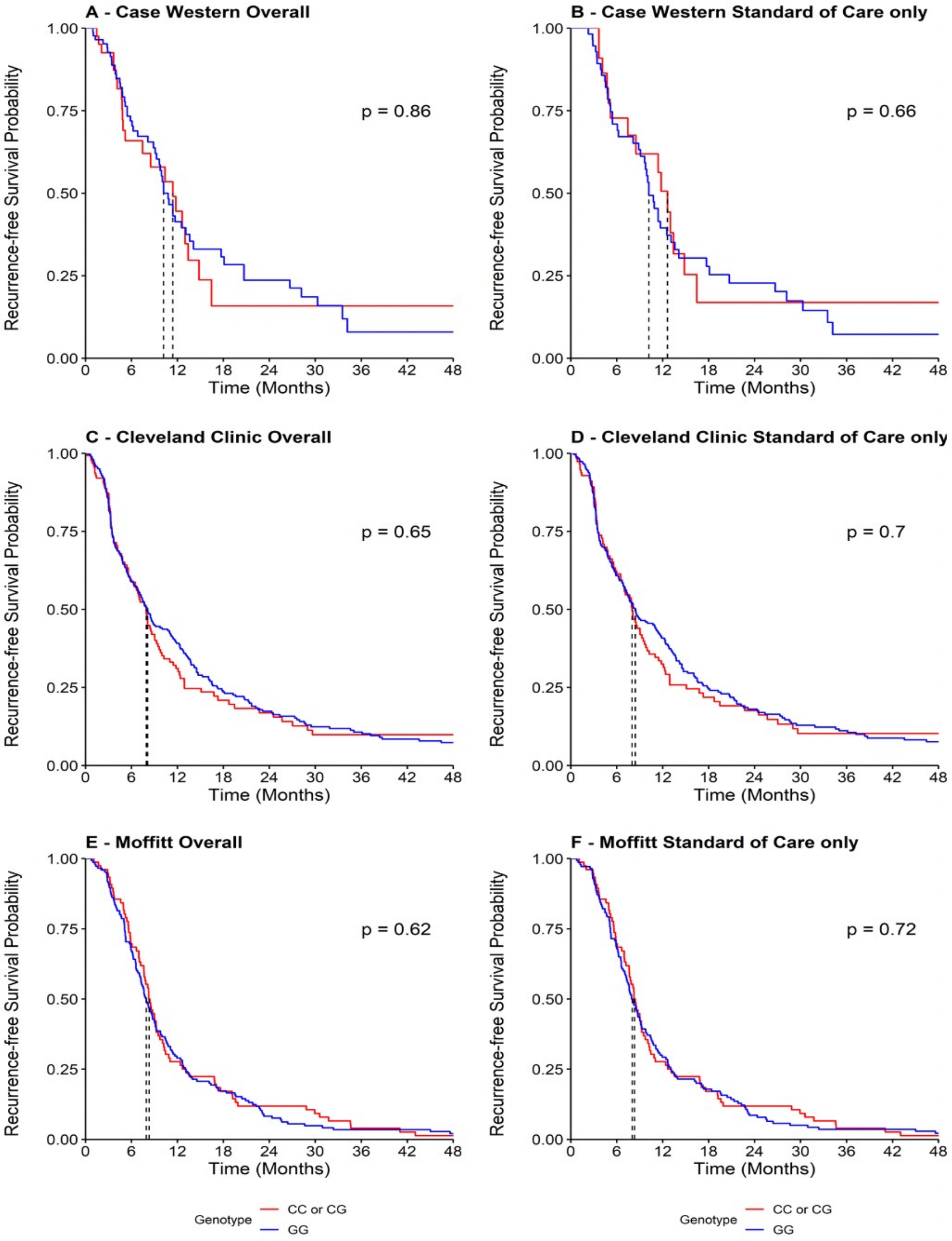

Supplemental Figure 4. Univariate analysis of clinical cohorts for *MIF* SNP rs755622 (progression-free survival). (**A-F**) Univariate analysis of recurrence-free survival comparing patients with the minor allele to patients homozygous for the major allele across all samples (left column) and then those who received full standard-of-care treatment protocol (right column), with log rank p value comparing each group provided to the right of each curve.

Supplemental Figure 5- ipa analysis of C/G (rs755622) vs homozygous dominant

A

IPA Analysis top 100 Genes (C/G)

Diseases or Functions Annotation	p-value
Synthesis of prostaglandin E2	5.96E-10
Synthesis of eicosanoid	7.53E-10
Innate immune response	7.83E-10
Systemic autoimmune syndrome	1.42E-09
Apoptosis of neutrophils	9.96E-09
Stimulation of myeloid cells	1.09E-08
Activation of cells	1.87E-08
Activation of macrophages	2.08E-08
Inflammation of gastrointestinal tract	2.79E-08
Inhibition of cells	3.72E-08
Stimulation of acute myeloblastic leukemia cells	4.52E-08
Chronic inflammatory disorder	5.42E-08
Activation of antigen presenting cells	5.93E-08
Rheumatoid arthritis	0.00000006
Rheumatic Disease	6.38E-08
Activation of neutrophils	0.000000098
Stimulation of granulocytes	0.000000101
Stimulation of cancer cells	0.00000011
Delay in apoptosis of neutrophils	0.00000011
Stimulation of phagocytes	0.000000127
Maturation of phagocytes	0.000000133
Activation of myeloid cells	0.000000142
Dermatitis	0.000000184
Cellular infiltration	0.000000209
Enteritis	0.000000236

B

IPA Analysis top 100 Genes (G/G)

Diseases or Functions Annotation	p-value
Hair/nail type ectodermal dysplasia type 4	0.00000855
Abnormal morphology of head	0.0000124
Morphology of head	0.0000125
Metabolism of histamine	0.0000256
Efflux of Ca2+	0.0000415
Development of interneurons	0.0000415
Cleft palate	0.0000442
Quantity of histamine	0.000076
Differentiation of lactotropes	0.0000851
Specification of neurons	0.0000938
Morphology of mouth	0.000201
Morphology of tongue	0.000207
Intercellular communication	0.000234
Morphology of posterior horn of the spinal cord	0.000237
Development of endocrine gland	0.000251
Abnormal morphology of central nervous system	0.00026
Morphology of central nervous system	0.00027
Abnormal morphology of nervous system	0.000281
Tachycardia	0.00036
Size of tongue	0.000379
Differentiation of nervous system	0.000397
Abnormal morphology of interneurons	0.000409
Development of jaw bone	0.000462
Differentiation of neurons	0.000462
Morphology of nervous system	0.000475

Supplemental Figure 5. Ingenuity pathway analysis of C/G vs homozygous dominant rs755622. Genes from the differential expression analysis of rs755622 minor allele vs major allele patients were ranked by log fold-change, and the top 100 genes increased with the minor allele and the top 100 decreased with the minor allele were subjected to Ingenuity Pathway Analysis. (**A-B**) Pathway description and p-value are shown for the top 25 pathways associated with the 100 genes.

Supplemental Figure 6- multiplex staining myeloid panel

A

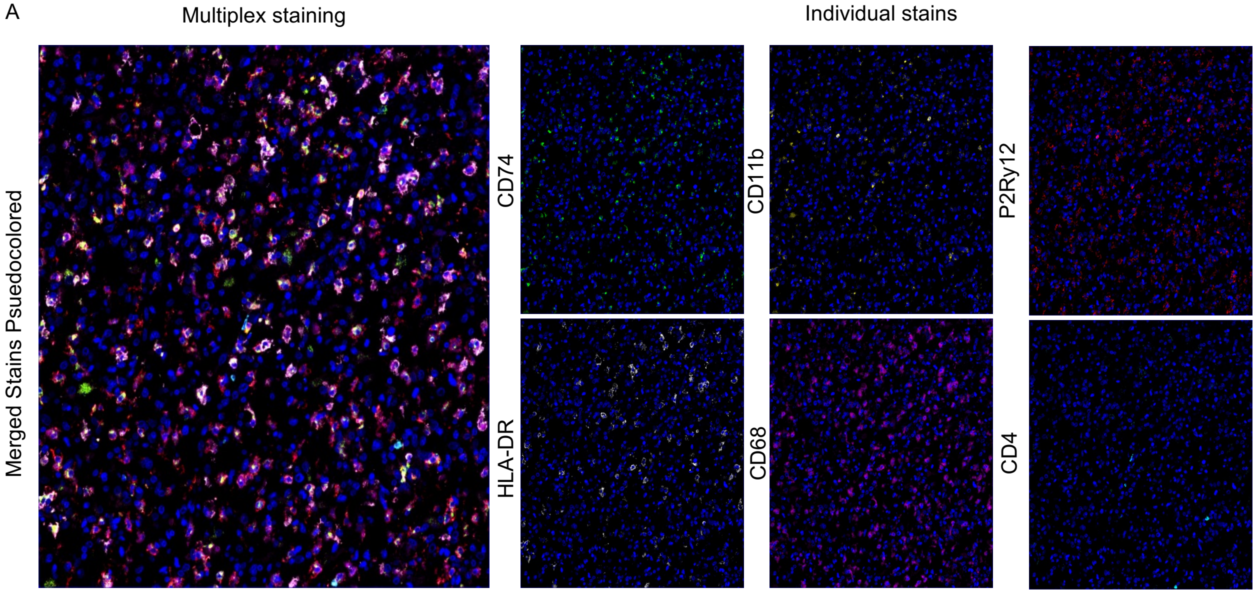

B

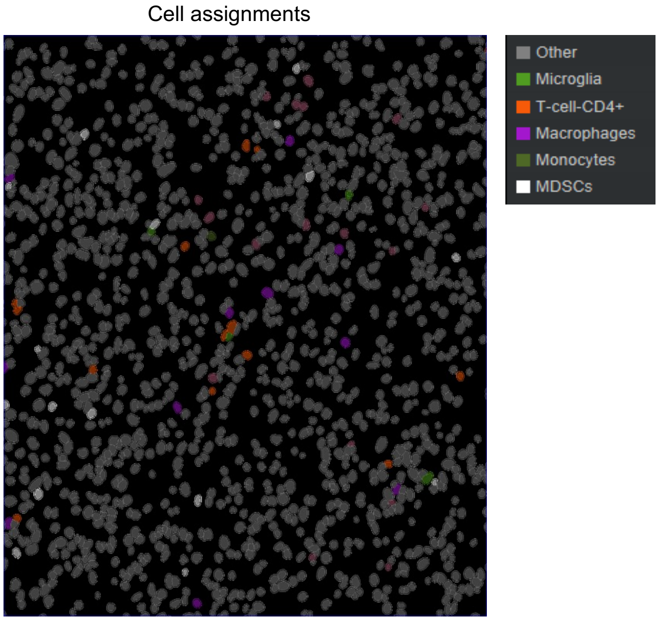

Supplemental Figure 6. Multiplex imaging myeloid cell identification (**A**) Representative images from a single region of one patient that contained all cell types of interest identified from the antibody panel. The left panel shows the merged image of all markers, while the right panels show single-channel image to highlight antibody specificity. (**B**) Cell assignments based on staining thresholds for markers are show pseudocoloring each cell type identifiable by the myeloid staining panel.

Supplemental Figure 7- TCGA_GBM analysis of the MIF SNP rs2096525 identifies increased immune signaling

A

MIF rs2096525
TCGA_GBM

Single Nucleotide Polymorphism	Rs2096525 CC=31,TC=91,TT=505
Major Allele (T)	87.8%
Minor Allele (C)	12.2%

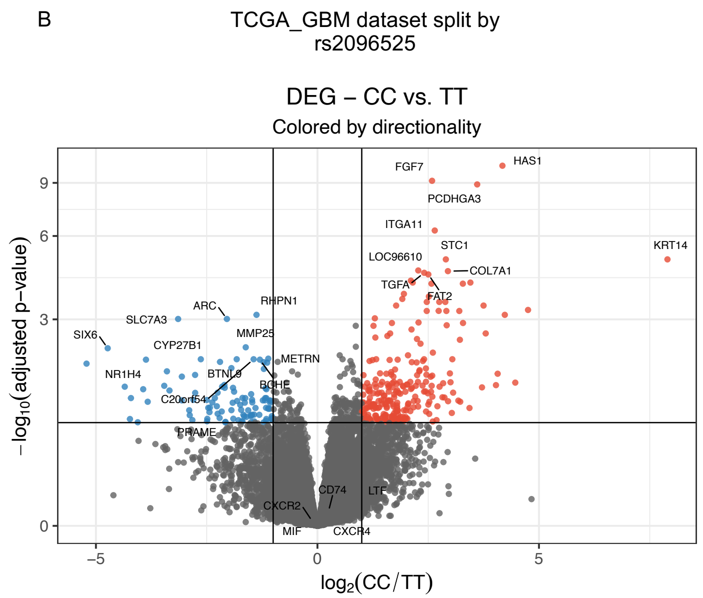

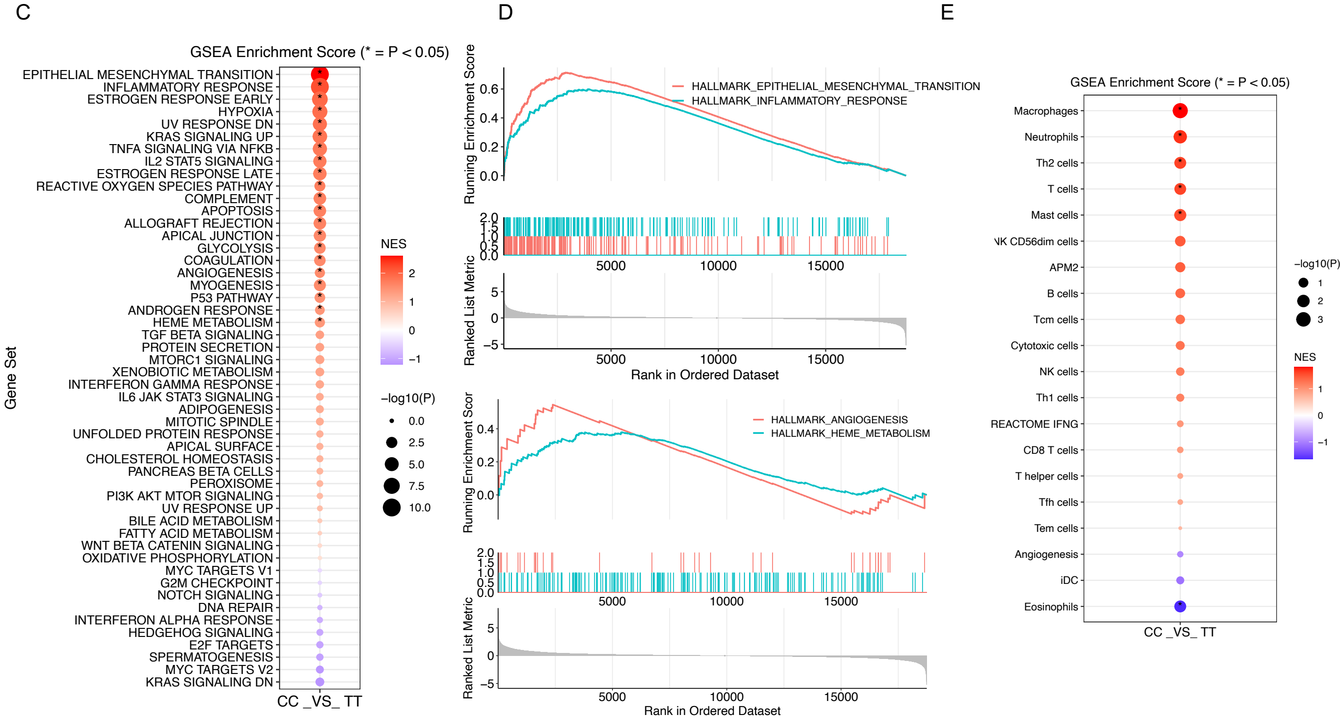

Supplemental Figure 7. TCGA_GBM analysis of the MIF SNP rs2096525 identifies increased immune signaling. (**A**) Analysis of TCGA_GBM whole-exome sequencing for rs2096525 identified 12.2% of patients with the minor allele. (**B**) The TCGA_GBM mRNA sequencing data and genotype information obtained from the whole-exome sequencing data (n=159) were used for differential expression analysis between patients with the minor allele (n=10) and patients homozygous for the major allele (n=113). Volcano plot represents deseq2 analysis of differential expression between the patients with the rs2096525 minor allele and patients homozygous for the major allele. (red= >1 log2FC & > 1 -log10 adjusted p-value, blue= <-1 log2FC & > 1 -log10 adjusted p-value) (**C**) GSEA analysis of Hallmark pathways from differential expression analysis of patients demonstrates increased pathways in rs2096525 SNP-containing patients. (**D**) Selected enriched top two pathways are shown for GSEA analysis, with ranked genes in each pathway for enrichment; the top two downregulated pathways also shown. (**E**) Pre-ranked gene lists of differential expression were utilized with the gene sets previously used for ssGSEA cell-type deconvolution. Deconvolution demonstrating an overall increase in macrophages, neutrophils, and T cell populations in samples from patients with the minor allele rs2096525 SNP.

Supplemental figure 8. Expression quantitative trait loci rs755622 using GTEX data portal

A

Tissue	Samples	NES	p-value	m-value
Esophagus - Gastroesophageal Junction	330	0.482	1.2e-7	1.00
Artery - Coronary	213	0.441	1.3e-4	1.00
Uterus	129	0.434	0.006	1.00
Artery - Aorta	387	0.418	2.7e-6	1.00
Cells - Cultured fibroblasts	483	0.408	6.1e-8	1.00
Stomach	324	0.399	4.2e-6	1.00
Artery - Tibial	584	0.381	8.5e-8	1.00
Minor Salivary Gland	144	0.376	0.01	1.00
Liver	208	0.370	0.006	0.990
Esophagus - Muscularis	465	0.367	4.0e-7	1.00
Adipose - Visceral (Omentum)	469	0.356	6.7e-6	1.00
Skin - Sun Exposed (Lower leg)	605	0.340	1.2e-6	1.00
Nerve - Tibial	532	0.337	7.8e-6	1.00
Vagina	141	0.329	0.02	0.995
Whole Blood	670	0.319	1.4e-6	1.00
Ovary	167	0.316	0.02	0.947
Prostate	221	0.302	4.3e-3	0.989
Adipose - Subcutaneous	581	0.300	2.9e-5	1.00
Small Intestine - Terminal Ileum	174	0.293	0.04	0.988
Thyroid	574	0.292	1.2e-4	1.00
Muscle - Skeletal	706	0.291	1.6e-6	1.00
Pancreas	305	0.278	2.7e-3	1.00
Esophagus - Mucosa	497	0.275	5.9e-4	1.00
Lung	515	0.263	6.7e-4	1.00
Adrenal Gland	233	0.260	0.007	1.00
Heart - Atrial Appendage	372	0.258	2.8e-3	1.00
Kidney - Cortex	73	0.252	0.3	0.768
Pituitary	237	0.244	0.03	0.984
Skin - Not Sun Exposed (Suprapubic)	517	0.240	1.3e-3	1.00
Colon - Sigmoid	318	0.229	0.02	0.968
Spleen	227	0.220	0.04	0.916
Brain - Substantia nigra	114	0.219	0.2	0.800
Brain - Spinal cord (cervical c-1)	126	0.212	0.2	0.871
Colon - Transverse	368	0.205	0.02	0.993
Breast - Mammary Tissue	396	0.200	0.02	0.924
Heart - Left Ventricle	386	0.194	0.01	0.940
Brain - Hippocampus	165	0.166	0.1	0.798
Cells - EBV-transformed lymphocytes	147	0.157	0.2	0.763
Brain - Frontal Cortex (BA9)	175	0.0837	0.4	0.271
Brain - Caudate (basal ganglia)	194	0.0468	0.6	0.0990
Brain - Cerebellum	209	-0.00789	0.9	0.00
Brain - Cortex	205	-0.0299	0.8	0.0150
Brain - Amygdala	129	-0.0805	0.5	0.0200
Brain - Cerebellar Hemisphere	175	-0.0877	0.3	0.00
Brain - Anterior cingulate cortex (BA24)	147	-0.105	0.4	0.00400
Brain - Nucleus accumbens (basal ganglia)	202	-0.139	0.1	0.00
Brain - Hypothalamus	170	-0.180	0.1	0.00
Brain - Putamen (basal ganglia)	170	-0.186	0.1	0.0240
Testis	322	-0.272	1.3e-6	0.00

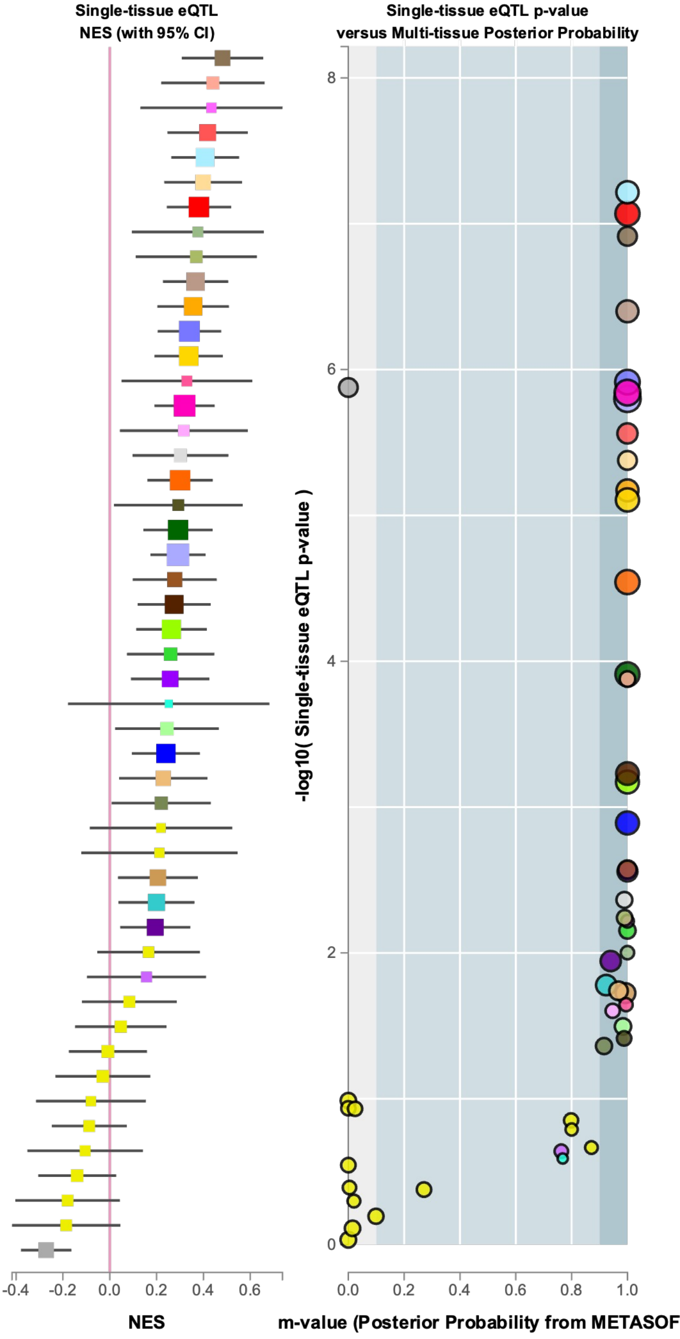

Supplemental Figure 8. GTEX database analysis of *MIF* and rs755622 as expression quantitative loci across tissues. Searching the GTEX database for rs755622 and its association across multiple tissues for a link with *MIF* expression shows significance in many tissues except central nervous system-related tissues.
